# Supplementary material for: Primary cilia deficiency in neural crest cells models anterior segment dysgenesis in mouse
Source: eLife. 2019 Dec 17;8:e52423. doi: 10.7554/eLife.52423 (PMC6946567; doi:10.7554/eLife.52423)
Supplement: Supplementary file 1. [file elife-52423-supp1.docx]

**Table. RT-qPCR primers**

Gene Forward Reverse Reference

*Axin2*5’-ctccccaccttgaatgaaga-3’5’-actgggtcgcttctcttgaa-3’(Grisanti, Revenkova, Gordon, & Iomini, 2016)

*Axin2* 5’-ctccccaccttgaatgaaga-3’ 5’-actgggtcgcttctcttgaa-3’ (Grisanti et al., 2016)

*B-catenin* 5’-cgcaagagcaagtagctgatattg-3’ 5’-cggaccctctgagccctagt-3’ (Grisanti et al., 2016)

*Cyclin D* 5’-aagtgcgtgcagaaggagat-3’ 5’-ttagaggccacgaacatgc-3’ (Grisanti et al., 2016)

*Dhh* 5’-TGGCATTGTGAGTTTCCTCCT-3’ 5’-AGCATGGACTTGGTTGGCTT-3’

*Foxc1* 5’-ttccctgccagtcagtctct-3’ 5’-gattctgttcgctggtgtga-3’

*Gapdh* 5’-cgtagacaaaatggtgaaggtcgg-3’ 5’-aagcagttggtggtgcaggatg-3’ (Grisanti et al., 2016)

*Gli1* 5’-tccgggcggttcctacg-3’ 5’-accatcccagcggcagtct-3’ (Grisanti et al., 2016)

*Hes1* 5’-GTCAACACGACACCGGACAA-3’ 5’-CCTTCGCCTCTTCTCCATGA-3’ (Grisanti et al., 2016)

*Hey1* 5’-GCTGAGATCTTGCAGATGAC-3’ 5’-CAACTTCGGCCAGGCATTCC-3’ (Grisanti et al., 2016)

*Ihh* 5’-CTGCAAGGACCGTCTGAACT-3’ 5’-TGGCTTTACAGCTGACAGGG-3’

*Lef1* 5’-tgaagcctcaacacgaacag-3’ 5’-gcccaggatctggttgatag-3’ (Grisanti et al., 2016)

*Maml1* 5’-GCACAGCGCGGTCATGGAGC-3’ 5’-GCGCTTGGCCTTGGCCTGGA-3’ (Grisanti et al., 2016)

*Pitx2* 5’-acgcgtacgcccacaaatg-3’ 5’-gctcgcaagcgaaaaatccta-3’ (Grisanti et al., 2016)

*Ptch1* 5’-acggggtcctcgcttacaaac-3’ 5’-tctcgtaggccgttgaggtagaa-3’ (Grisanti et al., 2016)

*Shh* 5’-TGTGTTCCGTTACCAGCGAC-3’ 5’-AGCGAGGAAGCAAGGATCAC-3’

*Smad2* 5’-gtctcttgatggccgtcttc-3’ 5’-cctgctgggaaatttgtgtt-3’ (Clement et al., 2013)

*Smad3* 5’-ttttcgtccagtctcccaac-3’ 5’-catctgggtgaggaccttgt-3’ (Clement et al., 2013)

*Smad4* 5’-tcgattcaaaccatccaaca-3’ 5’-gccctgaagctatctgcaac-3’ (Clement et al., 2013)

*Smad7* 5’-gcattcctcggaagtcaaga-3’ 5’-aggggccagataattcgttc-3’ (Clement et al., 2013)

*Tgfbr1* 5’-attgcagacttgggacttgc-3’ 5’-agaacagcgtcgagcaattt-3’ (Clement et al., 2013)

*Tgfbr2* 5’-ttcacagggacctcaagagc-3’ 5’-aacgactccacgttttccag-3’ (Clement et al., 2013)

Primers for *Dhh*, *Foxc1*, *Ihh* and *Shh* were designed with BLAST (<https://blast.ncbi.nlm.nih.gov/Blast.cgi>).

**References**

Clement, C. A., Ajbro, K. D., Koefoed, K., Vestergaard, M. L., Veland, I. R., Henriques de Jesus, M. P., . . . Christensen, S. T. (2013). TGF-beta signaling is associated with endocytosis at the pocket region of the primary cilium. *Cell Rep, 3*(6), 1806-1814. doi:10.1016/j.celrep.2013.05.020

Grisanti, L., Revenkova, E., Gordon, R. E., & Iomini, C. (2016). Primary cilia maintain corneal epithelial homeostasis by regulation of the Notch signaling pathway. *Development, 143*(12), 2160-2171. doi:10.1242/dev.132704
